# ﻿Description of two new species of freshwater leeches of the genus *Helobdella* (Hirudinea, Glossiphoniidae) from Mexico, with a redescription of *Helobdella socimulcensis* (Caballero, 1931)

**DOI:** 10.3897/zookeys.1261.162279

**Published:** 2025-11-26

**Authors:** Gerardo Torres-Carrera, Lucas Duarte-De Lima, Alejandro Oceguera-Figueroa

**Affiliations:** 1 Posgrado en Ciencias Biológicas, Universidad Nacional Autónoma de México, Avenida Universidad 3000, Ciudad Universitaria, Coyoacán, Ciudad de México, C.P. 04510, Mexico; 2 Laboratorio de Helmintología, Departamento de Zoología, Instituto de Biología, Universidad Nacional Autónoma de México, Ciudad Universitaria, Copilco, Coyoacán, A.P. 70-153, Ciudad de México, C.P. 14510, Mexico; 3 State University of Maringá UEM, Graduate Program in Comparative Biology, Av. Colombo, 5790 - Zona 7, Maringá – PR, 87020-900 Maringá, Paraná, Brazil

**Keywords:** Freshwater leeches, Glossiphoniiformes, Mexico, new species, parental care

## Abstract

Freshwater leeches of the genus *Helobdella* Blanchard, 1896 (Annelida: Clitellata) are widely distributed in the New World, with most of the species occurring in the neotropics and a single species native to Europe. Species of the genus are characterized by the presence of an eversible proboscis, dorsoventrally flattened body, presence of a single pair of eyespots, and a preference for feeding on the hemolymph of aquatic invertebrates. In this study, two new species of *Helobdella* are described based on specimens collected in Mexico, *Helobdella papilloprocta***sp. nov.** and *Helobdella gulloae***sp. nov.** These specimens were preliminarily identified as *H. socimulcensis* (Caballero, 1931), but detailed morphological and molecular analyses confirmed their status as separate species. In addition, a neotype for *Helobdella socimulcensis* is designated, and a redescription of the species is provided based on specimens from the type locality: Xochimilco, Mexico City, Mexico. Finally, *H. austinensis* in Nuevo León and *H. europaea* in Morelos, Mexico are reported for the first time, representing new records for the country. In total, ten species of *Helobdella* are known from Mexico.

## ﻿Introduction

The genus *Helobdella* Blanchard, 1896 (Annelida: Clitellata) includes proboscis-bearing freshwater leeches characterized by a dorsoventrally flattened body, gonopores separated by a single annulus, a single pair of cephalic eyespots, absence of esophageal organs (bacteriomes), and a feeding preference for the hemolymph of aquatic invertebrates (Govedich and Davies 1998; Siddall and Borda 2003). The genus *Helobdella* is the most speciose genus of Hirudinea with ~80 described species, with the majority of them distributed in the New World, particularly in the Neotropics (Christoffersen 2009; Magalhães et al. 2021). *Helobdella stagnalis* (Linnaeus, 1758), the type species of the genus, is widely distributed in Europe and is likely the only species to occur naturally outside the New World (Iwama and Arruda 2016; Saglam et al. 2018). In contrast, several species of *Helobdella* have been introduced to other continents, including Africa, Oceania, and Europe (Govedich and Davies 1998; Siddall and Budinoff 2005; Reyes-Prieto et al. 2014).

Sawyer (1986) subdivided the genus *Helobdella* into two mayor groups or series: the “*stagnalis*” series, comprising species with a chitinous nuchal scute located on the anterior dorsal surface, and the “*triserialis*” series, which includes species lacking this chitinous scute and bearing multiple pigmented longitudinal stripes along the dorsal surface. Previous phylogenetic analyses supported the monophyly of both series, however, subsequent studies incorporating additional species, many of which were previously assigned to separate genera that now are synonymized with *Helobdella*, have complicated the diagnosis of both series (Siddall and Borda 2003). In general terms, the taxonomy of *Helobdella* species is based on the position and shape of eyespots; the presence, absence, and arrangement of dorsal papillae and pigmented stripes as well as in internal traits including the number and size of salivary glands, gastric caeca and testisacs (see Marchese et al. 2020).

In Mexico, six species of *Helobdella* have been documented: *H. atli* Oceguera-Figueroa & León-Règagnon, 2005, *H. elongata* (Castle, 1900); *H. adiastola* Ringuelet, 1972; *H. temiscoensis* Salas-Montiel, Phillips, Pérez-Ponce de León & Oceguera-Figueroa, 2014; *H. socimulcensis* (Caballero, 1931), and *H. virginiae* Oceguera-Figueroa, 2007 (Oceguera-Figueroa et al. 2010; Salas-Montiel et al. 2014). In addition to these, two potentially new species have been identified through mitochondrial DNA sequence analyses, one closely resembling *H. socimulcensis* and the other *H. elongata* (see Tessler et al. 2018).

The “*triserialis*” series comprises at least ten species distributed across North and South America (Siddall and Borda 2003). *Helobdella socimulcensis*, originally described from Xochimilco Lake in Mexico City, is considered the only Mexican species of this complex. In addition to *H. socimulcensis*, two closely related forms from the Central Mexican plateau have been described: *H. conchata* Caballero, 1933 from Cuautla, Morelos and *H. moorei* Caballero, 1941, from León, Guanajuato. However, molecular and morphological analyses failed to differentiate specimens from these three nominal species collected at their respective type localities (Oceguera-Figueroa et al. 2010), thus supporting *H. socimulcensis* as the only valid species.

During an ongoing project focused on the characterization of the Mexican diversity of leeches, specimens morphologically similar to *H. socimulcensis* were collected in Chiapas, and Veracruz, Mexico. Detailed morphological comparisons of these specimens revealed notable morphological differences with the specimens from Xochimilco, Mexico City, the type locality of the species. These findings, together with the evidence provided by DNA sequences, clearly indicate the presence of two previously undescribed species. Due the need for a thorough comparison with *H. socimulcensis*, a redescription of that species is also provided. Furthermore, since Caballero (1931) did not designate a holotype or paratypes and none of the original material for this species remains, we herein designate a neotype for *Helobdella socimulcensis*.

## ﻿Materials and methods

### ﻿Collection of samples

Specimens examined in this study were collected between February 2014 and November 2024 (see Table 1). Specimens were found in freshwater habitats, attached to submerged rocks, plant roots, and bivalve shells, and were collected by hand. Leeches were maintained in plastic containers filled with water from the original habitat and transported to the laboratory for further analysis.

**Table 1.** Metadata associated with the specimens used in the present study. Numbers in bold denote newly generated sequences. CNHE = Colección Nacional de Helmintos.

Taxon	Locality	CNHE catalog number	GenBank accession number				Reference	GenSeq Nomenclature
			*cox*1	ND1	ITS	28S rRNA		
*Haementeria officinalis*	Michoacan, Mexico		JN850906	JN850952	JN850928	JN850891	Oceguera-Figueroa 2012	
*Helobdella adiastola*	Ejido Rosendo salazar, Chiapas, Mexico	11446, 11465	MK354140	**PX391404**	**PX380937**		**This study**	Genseq 4
*Helobdella adiastola*	Laguna Escondida, Los Tuxtlas, Veracruz, Mexico	11054	MK354139	**PX391403**	**PX380936**		**This study**	Genseq 4
*Helobdella atli*	Totolcingo, Tlaxcala, Mexico		HQ179851	**PX391395**	**PX380935**	**PX375459**	Oceguera-Figueroa et al. 2010; **this study**	Genseq 3
*Helobdella austinensis*	Shoal Creek, Austin, Texas, USA		DQ995306				Bely and Weisblat 2006	
*Helobdella austinensis*	Austin, Texas, USA		DQ995307				Bely and Weisblat 2006	
*Helobdella austinensis*	Shoal Creek, Austin, Texas, USA		DQ995310				Bely and Weisblat 2006	
*Helobdella austinensis*	Austin, Texas, USA		DQ995308				Bely and Weisblat 2006	
*Helobdella austinensis*	Shoal Creek, Austin, Texas, USA		DQ995309				Bely and Weisblat 2006	
*Helobdella austinensis*	Austin, Texas, USA		KC812736				Kutschera et al. 2013	
*Helobdella austinensis*	Rio San Juan, Nuevo Leon, Mexico		**PX366692**				**This study**	Genseq 4
			**PX366693**					
			**PX366694**					
			**PX366695**					
*Helobdella austinensis*	Balneario las Lajas, Veracruz, Mexico	12857	**PX366696**				**This study**	Genseq 4
*Helobdella austinensis*	Balneario Florida, Gómez Farías, Tamaulipas	12858	**PX366697**				**This study**	Genseq 4
*Helobdella austinensis*	Sombreretillo, Nuevo Leon, Mexico	11672	**PX366698**	**PX391402**	**PX380944**	**PX375460**	**This study**	Genseq 4
*Helobdella elongata*	California, USA		AF329045	AF329068			Siddall and Borda 2003	
*Helobdella eriensis*	Lake Erie, Toledo, Ohio, USA		MN071313				Saglam et al. 2018	
*Helobdella europaea*	Berkeley, California, USA		DQ995304				Bely and Weisblat 2006	
*Helobdella europaea*	Taipei, Taiwan		FJ000349				Lai et al. 2009	
*Helobdella europaea*	Temixco Morelos, Mexico	11673	**PX366685**	**PX391396**	**PX380938**	**PX375458**	**This study**	Genseq 4
*Helobdella europaea*	Castellon, Valencia, Spain		KC904242				Reyes-Prieto et al. 2014	
*Helobdella europaea*	Schobbach, Freiburg, Germany		AY576008				Pfeiffer et al. 2004	
*Helobdella europaea*	New Zealand		AY856049				Siddall and Budinoff 2005	
*Helobdella europaea*	Australia		AF329052				Siddall and Borda 2003	
*Helobdella europaea*	South Africa		AY856047				Siddall and Budinoff 2005	
*Helobdella farmeri*	California, USA		OR075101				Kutschera 2023	
*Helobdella fusca*	Wild Goose Lake, Michigan, USA		AF329038	AF329061			Siddall and Borda 2003	
*Helobdella gulloae* sp. nov.	Laguna escondida, Los Tuxtlas, Veracruz, Mexico	11133	**PX366688**	**PX391398**	**PX380939**	**PX375456**	**This study**	Genseq 2
*Helobdella lineata*	Michigan, USA		AF329039	AF329062			Siddall and Borda 2003	
*Helobdella modesta*	Connecticut, USA		JF319993	JF326527			Richardson et al. 2017	
*Helobdella octatestisaca*	Taipei, Taiwan		FJ000343				Lai et al. 2009	
*Helobdella papillata*	Virginia, USA		AF329046	AF329069			Siddall and Borda 2003	
*Helobdella transversa*	Michigan, USA		AF329044	AF329067			Siddall and Borda 2003	
*Helobdella papilloprocta* sp. nov.	Ejido Rosendo Salazar, Chiapas Mexico	11671, 11132	**PX366689**	**PX391399**	**PX380941**		**This study**	Genseq 2
*Helobdella papilloprocta* sp. nov.	Ejido Rosendo Salazar, Chiapas Mexico		**PX366690**	**PX391400**			**This study**	Genseq 2
*Helobdella papilloprocta* sp. nov.	Ejido Rosendo Salazar, Chiapas Mexico		**PX366691**		**PX380940**	**PX375457**	**This study**	Genseq 2
Helobdella aff. robusta	Sacramento, California, USA		DQ995300				Bely and Weisblat 2006	
Helobdella aff. robusta	Sacramento, California, USA		DQ995301				Bely and Weisblat 2006	
*Helobdella robusta*	Sacramento, California, USA		AF178680	AF178680			Siddall and Borda 2003	
*Helobdella socimulcensis*	Xochilmilco, Mexico City, Mexico		DQ995311				Bely and Weisblat 2006	
*Helobdella socimulcensis*	Xochilmilco, Mexico City, Mexico	11669, 11670	**PX366686**	**PX391397**	**PX380942**	**PX375455**	**This study**	Genseq 3
*Helobdella socimulcensis*	Guanajuato, Mexico	5561	HQ179870				Oceguera-Figueroa et al. (2010)	
*Helobdella socimulcensis*	Cuautla, Morelos, Mexico		HQ179872				Oceguera-Figueroa et al. (2010)	
*Helobdella socimulcensis*	Queretaro, Mexico	5563	HQ179868				Oceguera-Figueroa et al. (2010)	
*Helobdella socimulcensis*	Hidalgo, Mexico	5560	HQ179869				Oceguera-Figueroa et al. (2010)	
Helobdella cf. socimulcensis	Jalisco, Mexico’’	5562	HQ179866				Oceguera-Figueroa et al. (2010)	
*Helobdella socimulcensis*	Camecuaro, Michoacan, Mexico		**PX366687**				**This study**	
Helobdella cf. socimulcensis	Emiliano Zapata, Chiapas, Mexico		MG821615				Tessler et al. 2018	
*Helobdella temiscoensis*	Temixco, Morelos, Mexico		HQ179861				Oceguera-Figueroa et al. (2010)	
*Helobdella triserialis*	Bolivia		AF329054	AF329077			Siddall and Borda 2003	
*Helobdella virginiae*	Laguna de Catemaco, Veracruz Mexico	12859	**PX366699**	**PX391401**	**PX380943**	**PX375461**	**This study**	Genseq 3

### ﻿Morphological procedures

Leech specimens were narcotized by gradually adding 70% ethanol in a Petri dish filled with water until movement ceased. Subsequently, specimens were fixed in 96% ethanol and stored in 70% ethanol until further examination. Leeches intended for molecular analyses were fixed directly in 99% ethanol. To examine internal morphology, representative specimens were slightly flattened between two glass slides and posteriorly fixed and stained with a mixture of Meyer´s paracarmine and hematoxylin, cleared with methyl salicylate for 5–10 min, and mounted on glass slides using Canada balsam, following the protocols of Lamothe-Argumedo (1997). External and internal features were examined and illustrated using a Leica EZ4 stereomicroscope (Leica Microsystems, Schweiz) and an Olympus IX81 microscope (Olympus Optical CO, LTD, Tokyo, Japan), respectively. All measurements are provided in mm. Somite numbering follows Moore (1898).

Selected specimens were processed for scanning electron microscopy (**SEM**) following standard protocols. Samples were dehydrated through a graded ethanol series, critical-point dried with CO2, sputter-coated with a mixture of gold-palladium, and mounted on metal stubs using silver paste. Specimens were examined using a Hitachi Stereoscan Model SU1510 SEM at 10 kV at the Laboratorio Nacional de Biodiversidad (**LANABIO**), Instituto de Biología, Universidad Nacional Autónoma de México (**IB-UNAM**), Mexico. All specimens were deposited in Colección Nacional de Helmintos (**CNHE**), IB-UNAM.

### ﻿Molecular procedures

Tissue samples from the caudal sucker of representative specimens were removed and used for genomic DNA extraction with the Invitrogen PureLink® Genomic DNA mini kit, following the manufacturer’s protocol. The following loci were amplified: mitochondrial cytochrome c oxidase subunit 1 (*cox*1), nicotinamide adenine dinucleotide dehydrogenase subunit I (*nad*1), and the nuclear internal transcribed spacers 1 and 2 (ITS1, ITS2), including the complete 5.8 rDNA, as well as a partial fragment of the large subunit of ribosomal RNA (28S). All the reactions were accomplished with the primers proposed by Folmer et al. (1994) for *cox*1, Light and Siddall (1999) for *nad*1, White et al. (1990) and Källersjö et al. (2005), for ITS and Whiting (2002) and Prendini et al. (2005) for 28S. Polymerase chain reactions (PCR) were performed in a total volume of 15 μl containing: 2 μl of DNA template, 0.2 μl of each primer, 3 μl of 5× reaction buffer, 0.1 μl of MyTaq DNA polymerase (Bioline Cat. BIO-21105) and 9.5 μl of DNase-free H2O. Amplification conditions followed protocols described by Oceguera-Figueroa et al. (2005) and de Carle et al. (2017).

PCR products were visualized by electrophoresis on agarose gels. Successful amplifications were purified using CentriSep columns (Thermo Fisher Scientific) with 96-well filter plates with Sephadex G-50 (Cytiva). Sequencing reactions used 0.4 µl BigDye Terminator v. 3.1 (Applied Biosystems), 2 µl Buffer 5x, 4 µl ddH2O, 1 µl of primer at 10 µM, and 3 µl purified PCR product for a total volume of 10 µl. Samples were purified using Sephadex G-50 (Cytiva), then 25 µl 0.5 mM EDTA were added to each reaction. Sequencing reactions were performed using an ABI-PRISM® 3100 Genetic analyzer (Applied Biosystems) at LANABIO, IB-UNAM. DNA Contigs were assembled and edited using Geneious v. 5.1.7 (Biomatters Ltd. Auckland, New Zealand). All newly generated sequences were deposited in GenBank (Table 1).

### ﻿DNA sequence alignments and phylogenetic analyses

DNA sequences of each locus were aligned independently in MAFFT (Katoh et al. 2019) using the default parameters. A concatenated dataset was assembled in MESQUITE v. 3.7 (Maddison and Maddison 2021). *Haementeria officinalis* de Filippi, 1849 was selected as outgroup, based on previous analyses (Light and Siddall 1999; Siddall and Borda 2003; Oceguera-Figueroa 2012; de Carle et al. 2017). The final matrix included 56 terminals and 3,211 aligned nucleotides.

Phylogenetic analyses of the concatenated dataset were conducted under the maximum likelihood (ML) and Parsimony (P) optimality criteria. ML tree search was performed in IQ-TREE v. 2.0 (Nguyen et al. 2015). The appropriate model of sequence evolution was selected using ModelFinder (Kalyaanamoorthy et al. 2017). Branch support was estimated with rapid bootstrap (Hoang et al. 2018) establishing automatic number of replicates. Each partition was specified in a separate file and called with -p function. The selected substitution models were: GTR+F+I+G4 for *cox*1, TN+F+I+G4 for *nad1*, TPM3+F+I for ITS and TPM3+F for 28S. Parsimony analyses were conducted in TNT (Goloboff et al. 2008) using New Technology search algorithms with 1,000 replicates. Branch support was calculated with 1,000 pseudoreplicates of bootstrap. Pairwise genetic distances of the *cox*1 dataset were calculated in MESQUITE v. 3.7 (Maddison and Maddison 2021) under the Kimura 2-parameter (K2P) model (Nei and Kumar 2000).

## ﻿Results

### ﻿Phylogeny and molecular analyses

**Figure 1.**
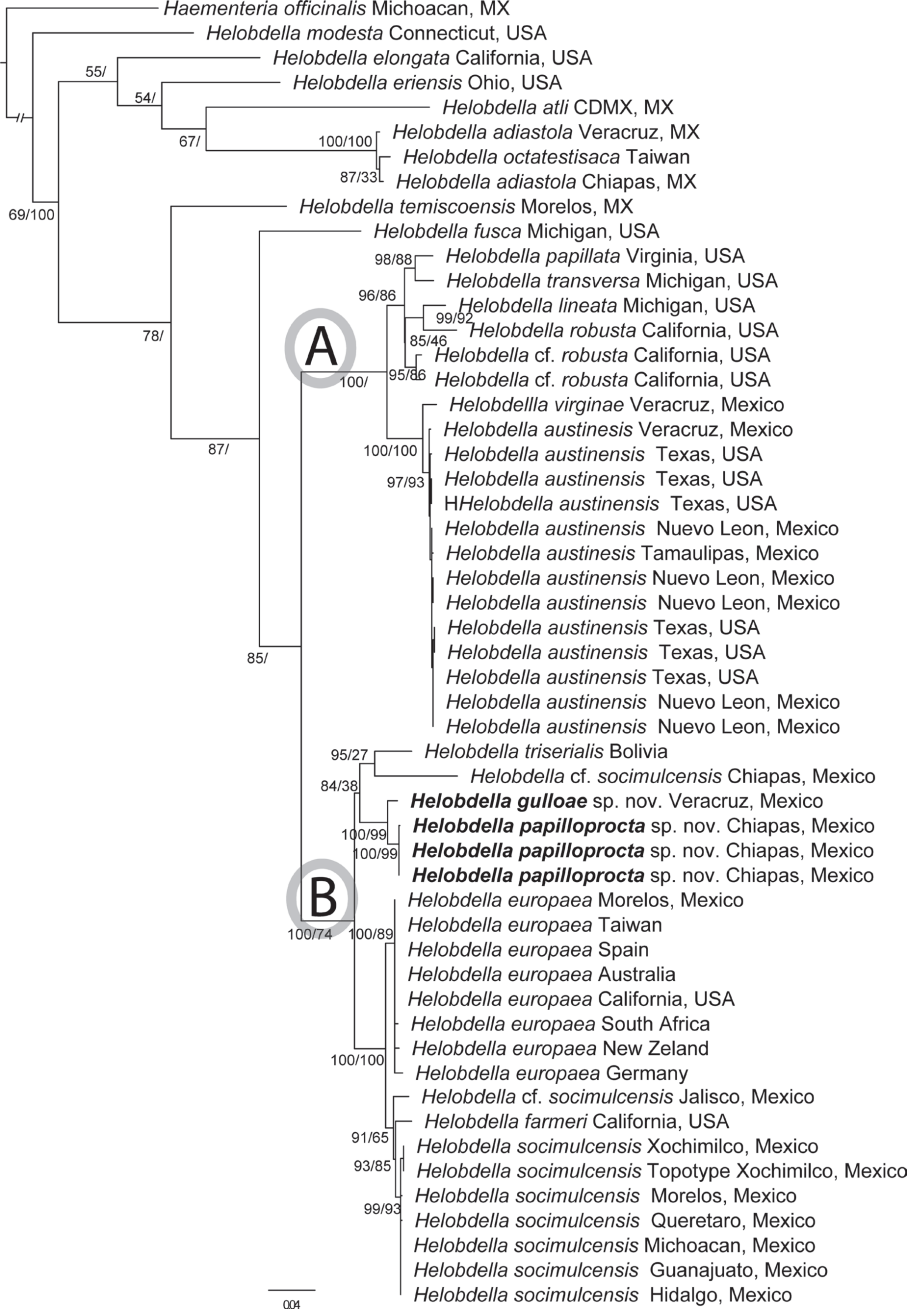
Maximum likelihood phylogenetic tree of species of *Helobdella* based on concatenated dataset of 3211 nucleotides. Values next to nodes indicate Maximum likelihood and Parsimony bootstrap values. New species described in this study are in bold. A and B indicate the two main species groups within the “*triserialis*” series.

Maximum likelihood phylogenetic analysis resulted in a tree with a log-likelihood of -22729.420 (Fig. 1). Parsimony analysis recovered four most parsimonious trees of 2484 steps each. The strict consensus tree of the parsimony analysis and the ML tree were largely congruent. Both analyses recovered the monophyly of the “*triserialis*” series sensu Sawyer 1986 (bootstrap support value of 87 in ML), while the “*stagnalis*” series was recovered as a paraphyletic assemblage. Species included in the “*triserialis*” series are characterized by the absence of a chitinous nuchal scute, in most of the species, and also the presence of multiple pigmented dorsal longitudinal stripes and metamerically arranged papillae (see Table 2).

**Table 2.** Key morphological characteristics of leeches in the *Helobdella triserialis* series sensu Sawyer (1986).

Taxon	Number of pairs of crop caeca	Number of pairs of testisacs	Number of dorsal brown stripes	Segment on which papillae begin	Papillae size with respect to width of annuli	Arrangement of dorsal white spots	Total number annuli	Venter coloration	Reference
*Helobdella austinensis* Kutschera, Langguth, Kuo, Weisblat & Shankland, 2013	6	6	16–20	apparently X	smaller	irregular	70	grey-brown	Kutschera et al. 2013
*Helobdella brasiliensis* (Weber, 1915)	6	6	absent	IX	larger	apparently metameric	70	stripes and spots dark brown	Ringuelet 1985
*Helobdella chaviensis* Iwama & Arruda, 2016	5	6	-	apparently IX	smaller	metameric	68	-	Iwama and Arruda 2016
*Helobdella europaea* (Kutschera, 1987)	5	5	14	X	larger	metameric	68	unpigmented to lightly pigmented	Kutschera 1985; Govedich and Davies 1998; Kutschera 2004
*Helobdella farmeri* Kutschera, 2023	6	6	8–14	absent	n/a	orthogonal	70	uniformly light-brown	Kutschera 2023
*Helobdella fusca* (Castle, 1900)	6	6	absent	absent	n/a	irregular	70	present	Castle 1900
***Helobdella gulloae* sp. nov.**	5	4	37	X–XIII	larger	metameric	69	cream with brown stripes	This study
*Helobdella lineata* (Verrill, 1874)	5	6	12–14	irregularly scattered	smaller	metameric	70	unpigmented	Sawyer 1972; Sawyer 2024
*Helobdella papillata* (Moore, 1906)	6	-	-	XIX–XX	larger	irregular	-	-	Sawyer 1972; Siddall and Borda 2003
***Helobdella papilloprocta* sp. nov.**	5	5	40	XII	larger	metameric	67	unpigmented	This study
*Helobdella robusta* Shankland, Bissen & Weisblat, 1992	5	-	-	-	apparently smaller	irregular	-	-	Shankland et al. 1992
*Helobdella socimulcensis* (Caballero, 1931)	5	5	34	X–XI	smaller	metameric	68	unpigmented	Caballero 1931
*Helobdella transversa* Sawyer, 1972	6	6	absent	absent	n/a	metameric form transverse bands	73	unpigmented	Sawyer 1972
*Helobdella triserialis* (Blanchard, 1849)	6	6	absent	apparently X	smaller	metameric	70	unpigmented	Ringuelet 1943
*Helobdella virginiae* Oceguera-Figueroa, 2007	6	6	30	XII	smaller	irregular	70	brown irregular spots	Oceguera-Figueroa 2007

Within the “*triserialis*” series, *Helobdella fusca* (Castle, 1900) from Michigan, USA was found as sister to the remaining species of the group. Two large clades were recovered: clade A includes *H. papillata* (Moore, 1906), *H. transversa* Sawyer, 1972, *H. lineata* (Verrill, 1874), and *H. robusta* Shankland, Bissen & Weisblat, 1992, from the USA; *H. virginiae* from Veracruz, Mexico; and multiple samples of *H. austinensis* Kutschera, Langguth, Kuo, Weisblat & Shankland, 2013 from Texas, USA and Nuevo León, México. Clade B includes *H. triserialis* (Blanchard, 1849) from Bolivia, H. cf. socimulcensis from Veracruz, Mexico, the invasive and worldwide-distributed *H. europaea* (Kutschera, 1985), *H. socimulcensis* from Mexico, and *H. farmeri* Kutschera, 2023 from California, USA. Finally, the two new species *H. papilloprocta* sp. nov. and *H. gulloae* sp. nov. are sister to each other, and together, sister to *H. triserialis* and H. cf. socimulcensis from Chiapas, Mexico, all together included in clade B.

## ﻿Taxonomy

### ﻿Order Hirudinida Siddall, Apakupakul, Burreson, Coates, Erséus, Gedlder Källersjö, & Trapido-Rosenthal, 2001

**Suborder Glossiphoniiformes Tessler and de Carle, 2018**

**Family Glossiphoniidae Vaillant, 1890**

#### ﻿Genus Helobdella

Taxon classificationAnimaliaHirudinidaGlossiphoniidae

Blanchard, 1896

A83E8983-ECE0-5D1D-8A65-931E969678BE

##### Type species.

*Helobdella stagnalis* (Linnaeus, 1758).

#### ﻿ Helobdella socimulcensis

Taxon classificationAnimaliaHirudinidaGlossiphoniidae

(Caballero, 1931)

6196F8F3-009D-5BCD-8E78-30F03ECAD9AB

Figs 2, 3, 4

Helobdella moorei Caballero, 1933 – León, Guanajuato; Mexico.
Helobdella conchata Caballero, 1941 – Cuautla, Morelos Mexico.
Helobdella triserialis var. lineata Ringuelet, 1943 – Xochimilco, Mexico City (sensu Ringuelet 1981).

##### Neotype designation.

Mexico • A stained adult, mounted in a slide. 11.5 length and 4.5 maximum width at somite XIX; Xochimilco, Mexico City; 19°17'21"N, 99°06'31"W; Collected by G.T-C. CNHE 12299.

##### Note.

According to Article 75.3 of the ICZN, a neotype is designated due to the absence or loss of the original type material. Caballero (1931) did not designate any specimen as part of the type series, and no specimens attributable to the original description of *Glossiphonia socimulcensis*, 1931 have been found in the CNHE or any other scientific collection. Furthermore, the nomenclatural act presented here is justified by the recognition of five lineages with specimens that exhibit morphological similarities to *H. socimulcensis*. In the absence of type material, meaningful comparisons are hindered. The neotype is designated from material collected from the type locality (Lake Xochimilco) and after ensuring a morphological correspondence with the original description. Finally, DNA sequences were obtained from specimens collected in the present study; these sequences have been validated and comply with the recommendations outlined by Kvist et al. (2010).

##### Material examined.

Mexico • Neotype and 20 adult specimens in total: 13 stored in ethanol, six mounted on slides and one processed for SEM; same data as for Neotype; CNHE 12298. Mexico • 116 ethanol-preserved adult specimens; same data as for Neotype; collected by GT-C between April and November 2021; CNHE 13037. Mexico • six adult specimens preserved in ethanol and one slide of an immature specimen; same data as for Neotype; Collected by Salas-Montiel and AO-F; CNHE 8860. Mexico • One adult specimen; same data as for Neotype; collected by AO-F on February 2003; CNHE 4705 (Oceguera-Figueroa 2007. Mexico • two adult specimens; Lago de Tecocomulco, Hidalgo; 19°51'57"N; 98°23'51"W; collected by AO-F on 21 August 2004 CNHE 4704. Mexico • “*Hellobdela conchata*” Holotype Cuautla, Morelos CNHE 1852, and 2 adult paratypes; same data as for Holotype; CNHE 1853.

##### Diagnosis.

Small leeches, 4–16 length and 1.5–9 width (*n* = 115). Dorsal surface with ~34 brown stripes. Midline longitudinal paired stripes of solid color extend from Va2 to the anus, separated by a distance approximately equal to that between eyespots; the stripes join each other at a2 of each somite, creating a chain-like appearance. Three dorsal rows of longitudinal, dark brown-tipped papillae on a2: one median and two lateral rows. In some specimens, an additional pair of marginal rows more prominent in the posterior third of the body. Papillae smaller than the width of individual annuli. Eyespots punctiform and well separated. Full body with 68 annuli. Esophagus slightly globular, spanning six annuli. Crop with five pairs of digitiform caeca, the last pair forming post-caeca. Testisacs in five intersegmental pairs.

##### Description.

**Figure 2.**
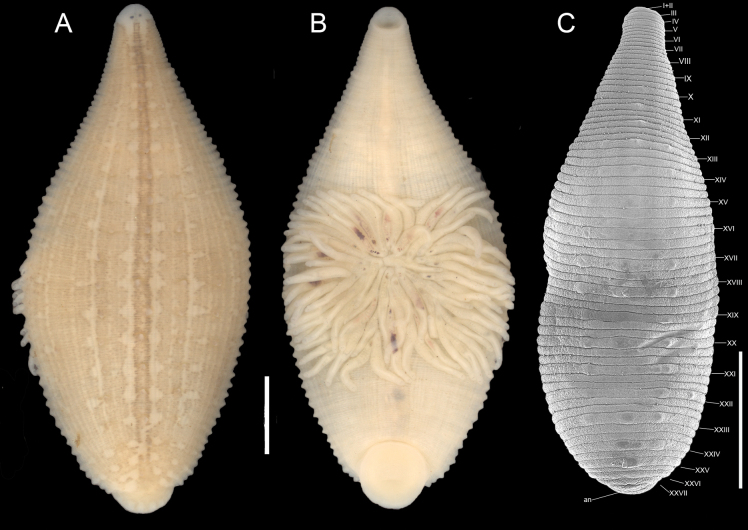
*Helobdella socimulcensis* from Xochimilco, Mexico. **A.** Dorsal view; **B.** Ventral view showing attached broods; **C.**SEM microphotograph showing somite numbering. Abbreviations: an = anus. Scale bar: 2 mm.

***External morphology*.** Body lanceolate, 3–16 length and 1.5–9 maximum width at somite XIX (*n* = 115). Ground color pale yellow or cream; dorsal preocular zone unpigmented (Fig. 2A–C). Dorsal surface with ~34 stripes of longitudinal brown stripes (visible in stained specimens). A pair of solid midline stripes, separated by a distance equivalent to the distance between eyespots, extends from Va2 to the anus at XXVII. The stripes join each other at a2 of each somite, creating a chain-like appearance. The remaining 32 stripes (16 pairs) are slender extend from Va2 or Va3 (annuli 6 or 7) and continue to the anus (Fig. 2A). Dorsum with three longitudinal rows of dark brown-tipped papillae located in a2 of each somite, one row median and two laterals. In some specimens, an additional pair of incomplete marginal rows may be present, papillae more prominent in the posterior third of the body, some papillae unpigmented (Fig. 2A). Median papillae row extends from X–XI to XXV–XXVI; lateral rows from XIII to XXVI (Fig. 2C). Papillae smaller than the width of an annulus. Dorsal white spots conspicuous, in three longitudinal rows metamerically arranged: one central and two laterals. White spots somewhat circular, approximately the size of an annulus and located in a2, beside lateral rows of papillae. Median row of white spots from VIII to XXVI; lateral rows from XII or XIII. Eyespots punctiform at IV a1/a2, well-separated. Anus dorsal, at XVII. Ventral surface whitish, without papillae. Mouth pore located at the anterior margin of the anterior sucker. Gonopores separated by a single annulus, male gonopore in XII a1/a2, female gonopore in XII a2/a3. In total, 68 annuli along the body. Annulation: I–II fused; III uni-annulate; IV bi-annulate; V–XXIV tri-annulate; XXV bi-annulate; XXVI and XXVII uni-annulate (Fig. 2C).

**Figure 3.**
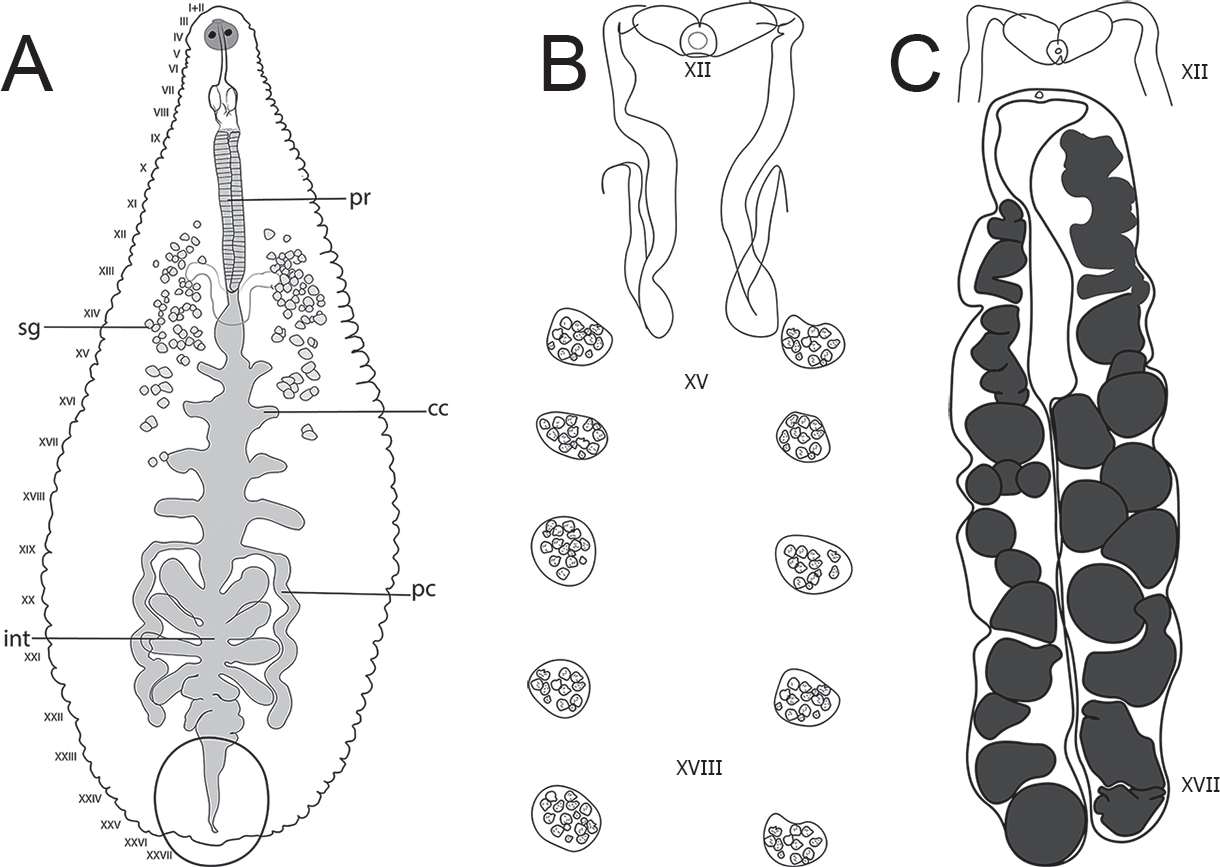
*Helobdella socimulcensis* from Xochimilco, Mexico City, Mexico. Internal morphology. **A.** Digestive system; **B.** Male reproductive system; **C.** Male and female reproductive systems. Abbreviations: Pr = proboscis; sg = salivary glands; cc = crop caeca; pc = post-caeca; int = intestine.

***Internal morphology*.** Proboscis straight, not curved. 1.95 total length, extending from IX to XIV when retracted. Salivary glands diffuse in the parenchyma, extending from XI to XVII. Salivary ducts connect to the base of the proboscis at XIV. Esophagus enlarged, slightly globular, chamber-like, from XIIIa3 to XV a1. Crop with five pairs of digitiform caeca, the first four pairs laterally directed, with the first pair at XV; the fifth pair forming sinuous post-caeca from XIX to XXIII (Fig. 3A). Intestine with four pairs of caeca, the first three pairs directed slightly anteriorly and last pair directed posteriorly. Male atrial cornua laterally directed. Vas deferens with a short descending loop, reaching the first pair of testisacs or XIV (Fig. 3B, C). Five pairs of testisacs present, first pair between XIV and XV. Ovisacs simple, elongate, and not folded, extending to XVII or XVIII (Fig. 3C).

**Figure 4.**
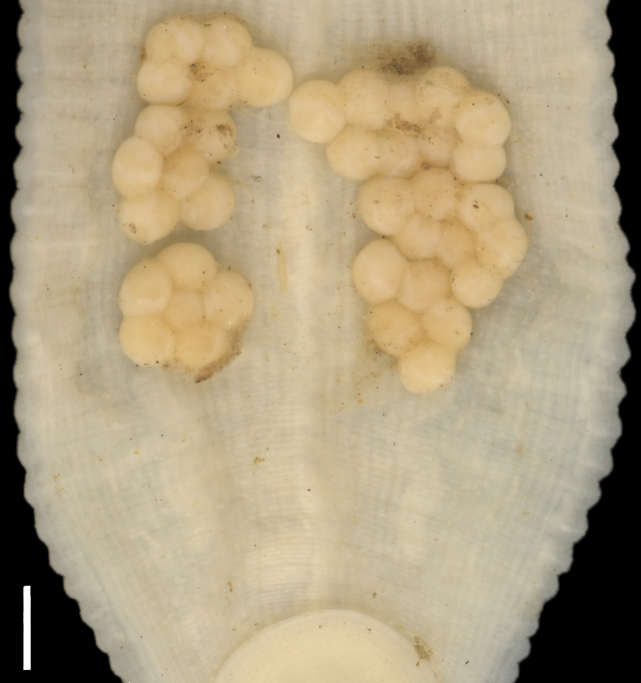
*Helobdella socimulcensis* from Xochimilco, Mexico. Ventral surface showing eggs attached. Scale bar: 500 μm.

***Reproductive information*.** No specimens with spermatophores attached to the body wall were observed at any time. In total, 18 of 116 specimens were found with five or six thin-walled cocoons attached to the ventral surface. The total number of eggs ranges from 20 to 100. The brooding area extended from XVIIa2 to XXa3 or XVIa3 to XXI (Fig. 4). Egg diameter 306–479 μm (373, *n* = 29). Some specimens carried up to 69 embryos attached to ventral surface (Fig. 2B), easily lost during manipulation and fixation. The highest proportion of gravid or brooding individuals were collected between May and July.

##### Remarks.

The newly collected specimens collected in Xochimilco exhibit morphological traits consistent with the original description of *H. socimulcensis*, with two differences: a smaller maximum body length (10 vs 15) and higher number of annuli (68 vs 65). Regarding the difference in the number of annuli, Caballero (1931) considered that eyespots were located in somite I instead IV a2. This difference in annuli numbering accounts for the apparent difference.

#### ﻿ Helobdella papilloprocta sp. nov.

Taxon classificationAnimaliaHirudinidaGlossiphoniidae

350992B6-4570-5AE8-AE41-F237A8B92167

https://zoobank.org/86713D6E-D6BB-4149-BC57-21412C4EFC70

Figs 5, 6, 7

##### Type material.

***Holotype*.** Mexico • adult; Rosendo Salazar, Municipio de Cintalapa, Chiapas; 16°28'14.696"N, 93°59'58.426"W, collected by GT-C and AO-F on April 2017; CNHE 12295. ***Paratypes*.** Mexico • 28 adult paratypes; same data as for Holotype; CNHE 11132.

##### Diagnosis.

Small leech, 5–9 length and 2.5–3.8 maximum width at somite XIX (*n* = 14). Dorsal surface with ~40 longitudinal brown stripes. Brown coloration present or fade in larger specimens. Midline paired stripes not joining. Three longitudinal rows of black-tipped papillae, one median and two lateral rows, with additional incomplete marginal rows in all specimens. Papillae larger than the width of annuli. A pair of dorsal papillae located beside the anus. Eyespots oval, well separated. Whole body with a total of 67 annuli. Esophagus short. Crop with five pairs of caeca, the last pair forming post-caeca. Testisacs in five intersegmental pairs.

##### Description.

Based on adult specimen (holotype), CNHE 12295 (stained), 7 length and 3.7 maximum width at somite XIX a3. Twenty-eight paratypes, CNHE11132, include 15 adult alcohol-preserved specimens, 10 flattened and stained specimens, and 3 specimens prepared for SEM.

**Figure 5.**
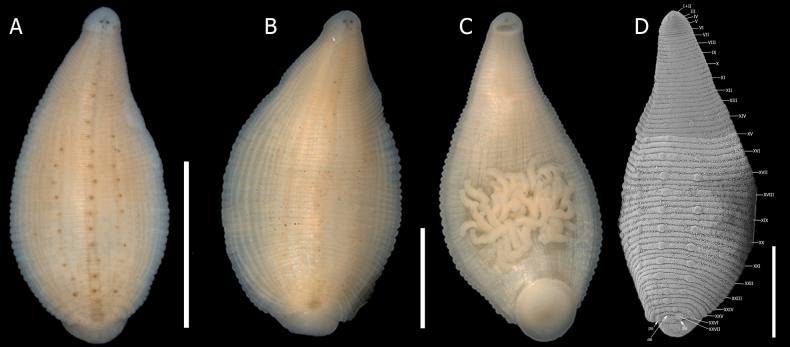
*Helobdella papilloprocta* sp. nov. from Ejido Rosendo Salazar, Cintalapa, Chiapas, Mexico. **A.** Dorsal view of a small specimen with conspicuous pigmentation; **B.** Dorsal view of a specimen with faded pigmentation; **C.** Ventral view of faded-pigmentation specimen with young leeches attached; **D.**SEM microphotograph showing somite numbering. Abbreviations: an = anus; pa = papillae. Scale bar: 2 mm

***External morphology*.** Body lanceolate to ovoid, 5–9 length and 2.5–3.8 maximum width at XIX. Ground color pale yellow or cream; dorsal preocular zone unpigmented. Dorsal surface with ~40 longitudinal stripes (visible in stained organisms). Patterns of metameric brown pigmentation and pigmented papillae, features otherwise characteristic of species in the “*triserialis*” series, are visible in some specimens. However, this pigmentation tends to fade in larger specimens (Fig. 5A, B). Paired midline stripes extend from annulus 5 (Va1) to the anus at XXVII and remain not joined. Additional brown stripes extend from annulus 6 or 7 (Va2 or Va3) to the anus.

Dorsal surface with three longitudinal rows of pigmented papillae, one median and two lateral rows; an additional pair of incomplete paramedial rows present in all specimens. Median row of papillae from XII to XXVI, smaller additional paired papillae may be present in somite XXI and continue posteriorly (Fig. 5D). Lateral rows from XIV and continue posteriorly. Papillae generally wider than the width of individual annuli. A pair of prominent papillae located adjacent the anus (Fig. 5D). White dorsal spots faint, circular and larger than the length of an annuli, arranged in two pairs of rows per side, located at each a2 adjacent to the papillae. White spots beside median papillae beginning at X and reaching XXVII, lateral white spots at XIV and reaching XIV. Eyespots cup-like at IVa2, well separated. Ventral surface white (Fig. 5C). Mouth pore located at the anterior margin of the anterior sucker. Gonopores separated by a single annulus (XII a2). Whole body with 67 annuli. Anus on XVII. Annulation: I–II fused; III uni-annulate; IV and V bi-annulate; VI to XXIV tri-annulate; XXV biannulate; XXVI and XXVII uni-annulate (Fig. 5D).

**Figure 6.**
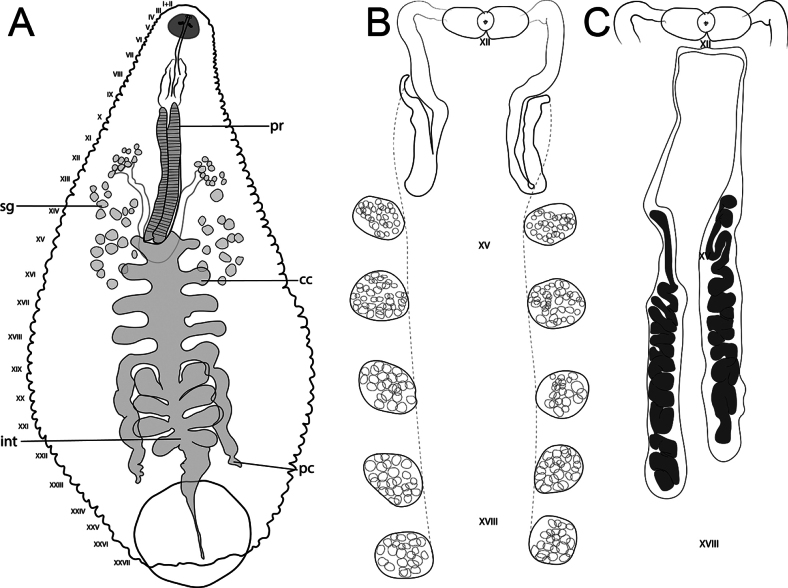
Internal morphology of a stained paratype specimen of *Helobdella papilloprocta* sp. nov. **A.** Digestive tract details; **B.** Male reproductive system; **C.** Female reproductive system. Abbreviations: pr = proboscis; sg = salivary glands; cc = crop caeca; pc = post-caeca; int = intestine.

***Internal morphology*.** Proboscis straight, not recurved, 1.8 length, extending from IX to XIV, or reaching XV when retracted. Salivary glands diffuse into parenchyma from XI to XVII; ductules not forming bundle, insert independently into base of proboscis. Esophagus short, located at XV a1. Crop with five pairs of digitiform caeca; the first four pairs laterally directed, fifth pair with a posterior sinuous path or post-caeca, from XIX to XXIII (Fig. 6A). Intestine with four pairs of digitiform caeca, the first three slightly oriented anteriorly, and last pair directed posteriorly. Five pairs of testisacs, first pair between XIV/XV (Fig. 6B), last pair between XVIII/XIX. Ejaculatory ducts with a short posterior loop reaching XIV and then with an anterior trajectory to XIII. Ovisacs simple, elongated, extending posteriorly to XVII or XVIII (Fig. 6C).

**Figure 7.**
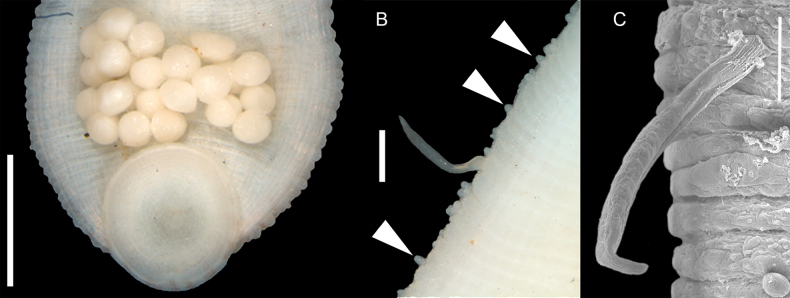
*Helobdella papilloprocta* sp. nov. **A.** Eggs attached to the ventral surface; **B.** Spermatophore attached to the body wall and epibionts are indicated with white arrows; **C.**SEM microphotograph of a spermatophore. Scale bars: 1 mm (**A**); 200 μm (**B**); 100 μm (**C**).

***Reproductive and ecological information*.** Specimens were found attached to the shells of the invasive Asian clam *Corbicula fluminea* (Müller, 1774). Eight of 21 collected leeches with eggs or young leeches attached to the ventral surface, carrying between 21 to 36 eggs or 51 young leeches (Figs 5C, 7A). Brooding area spans ~15 annuli, from XVIII a3 to XXIII (Fig. 7A). Egg diameter 238–428 μm (322, *n* = 7). Spermatophores observed in three of 21 leeches, attached to the body wall, between annuli 24–27 (Fig. 7B, C). Each spermatophore ~350 μm length and 40 μm width. Spermatophores with a broad proximal portion attached on body wall of the recipient specimen, a narrow middle region or neck and distal portion with two horn-like tubes. Almost all leech specimens with epibionts (ciliates) attached to the body wall (Fig. 7B).

##### Etymology.

The specific epithet refers to the presence of a distinctive pair of papillae located adjacent to the anus.

##### Remarks.

The morphological characteristics of *H. papilloprocta* sp. nov. are consistent with those diagnostic of the genus (Sawyer 1986; Siddall and Borda 2003); including the dorsoventrally flattened body, gonopores separated by a single annulus, one pair of cephalic eyespots, and the absence of esophageal organs (bacteriomes). The presence of multiple, longitudinal dorsal stripes and metamerically arranged papilla, particularly prominent in smaller leeches, clearly support its inclusion in the “*triserialis*” series sensu Sawyer (1986).

Species of the “*triserialis*” series can be differentiated from *H. papilloprocta* sp. nov. by distinct patterns of dorsal pigmentation and papillae distribution. *Helobdella papillata* has three rows of papillae restricted to the posterior third of dorsum (XIX or XX), while in *H. papilloprocta* sp. nov., dorsal rows of papillae begin at XII. Additionally, *H. papillata* lacks dorsal pigmentation. In *H. lineata*, dorsal papillae are irregularly scattered, while in *H. papilloprocta* sp. nov. dorsal papillae are regularly arranged. *Helobdella transversa* lacks both longitudinal pigmented stripes and papillae (Sawyer 1972; Light and Siddall 1999), clearly contrasting with *H. papilloprocta* sp. nov. *Helobdella lineata* exhibits 12–14 longitudinal stripes on the dorsal surface, whereas *H. papilloprocta* sp. nov. display ~40. *Helobdella fusca* and *H. virginiae* are characterized by irregularly arranged dorsal spots, clearly contrasting with the pattern observed in *H. papilloprocta* sp. nov. (Table 2).

*Helobdella papilloprocta* sp. nov. is morphologically and phylogenetically (see below) closely related to *H. socimulcensis*, *H. gulloae* sp. nov., *H. farmeri* Kutschera, 2023, *H. europaea*, and *H. triserialis*. Differentiation based on the morphological basis may be complex. However, the presence of two prominent papillae adjacent to the anus characteristic of *H. papilloprocta* sp. nov. appears to be a consistent and reliable character not present in other species of the genus.

#### ﻿ Helobdella gulloae sp. nov.

Taxon classificationAnimaliaHirudinidaGlossiphoniidae

9FD084D8-8D7A-5078-9F17-2A2E967956C1

https://zoobank.org/2DB933D1-8358-49AB-ABBD-C020406E5D99

Figs 8, 9

##### Type material.

***Holotype*.** Mexico • adult; Laguna Escondida, Municipio de San Andres, Tuxtla, Veracruz; 18°35'39.5761"N, 95°04'55.6140"W; collected by GT-C and AO-F on April 2017; CNHE 11133. ***Paratypes*.** Mexico • 6 adults paratypes; same data as for Holotype; CNHE 12296.

##### Other material.

Mexico • Four stained adults; Nanciyaga, Catemaco, Veracruz; 18°26'50.1169"N, 95°04'09.2788"W Collected by GT-C and FR-E in November 2021. CNHE 12297.

##### Diagnosis.

Small leech, 4.5–8 length and 2–2.5 maximum width at somite XIX (*n* = 6). Dorsum with ~37 longitudinal brown stripes. A pair of midline brown stripes originates near the eyespots until XVI, then fusing forming a single, broad stripe that reach XXI. Beyond this somite, the stripe is interrupted by square white spots, from XXI a3 to the posterior end. Three longitudinal rows of black-tipped papillae present; additional, incomplete rows occasionally observed near the body margins. Papillae slightly wider than individual annuli. Eyespots semi-oval. Whole body with total of 69 annuli. Esophagus short, extending one or two annuli in length. Crop with five pairs of caeca, last pair forming post-caeca. Intestine with four caeca. Testisacs in four intersegmental pairs.

##### Description.

Description based on holotype (stained), 9.6 length and 2.3 maximum width at somite XIX a2, and six paratypes, five stained and one processed for SEM.

**Figure 8.**
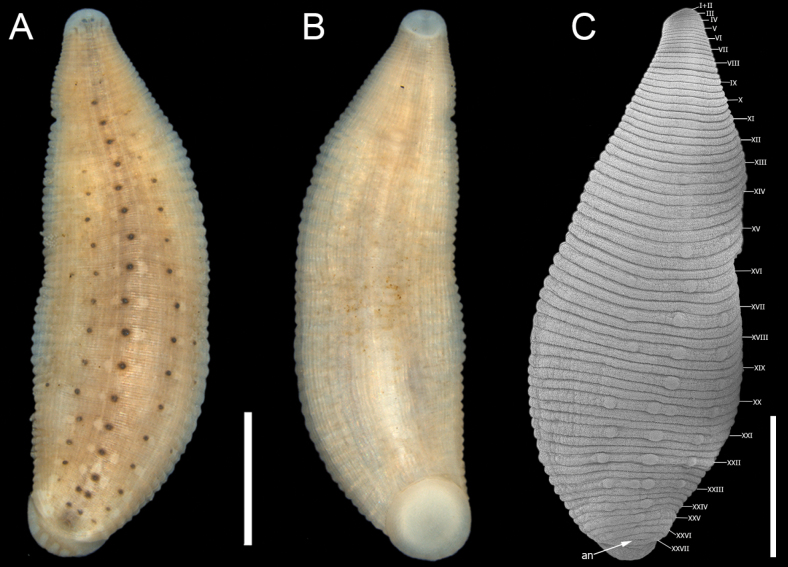
*Helobdella gulloae* sp. nov. **A.** Dorsal view; **B.** Ventral view; **C.**SEM microphotograph showing somite numbering. Abbreviations: an = anus. Scale bar: 2 mm.

***External morphology*.** Body slightly lanceolate, 4.5–8 length and 2–2.5 maximum width at somite XIX. Ground color pale yellow or cream; dorsal preocular zone unpigmented (I + II to Va2) except for the anterior end of the dorsal midline stripes that extend to reach the eyespots (Fig. 8A). Dorsal surface with the characteristic brown longitudinal striping pattern of species of the “*triserialis*” series, with ~37 brown stripes visible in stained specimens. A pair of longitudinal midline stripes from IVa3 and fuse into a single, broad stripe from XVI to the anus; this band is interrupted by square-shaped white spots located at each a3/a1 from XXII to the posterior end. The remaining pairs of dorsal brown stripes extend from IVa3 to the anus. Three rows of dorsal papillae heavily pigmented on a2. Additional lateral may occur though these are typically incomplete. Papillae slightly exceed the width of individual annulus. Midline row of papillae extends from X, with conspicuous papillae evident from XII or XIII to XXVI (Fig. 8C). Lateral rows of papillae begin at XIV to XXV. White spots conspicuous, square to circular in shape, and smaller than the width of individual annulus; arranged in two paired rows on each side of the body, located on a2, flanking each papilla. White spots flanking central papillae begin at V or VI, and extend to XXVI, lateral rows of white spots extend from XII to XXVII. Eyespots semioval, at IVa1+a2, well-separated. Anus on XVII, typically surrounded by black pigment. Ventral surface ground cream-colored, with faint, thin longitudinal brown stripes (Fig. 8B). Oral sucker white; mouth located at the anterior margin. Gonopores separated by a single annulus (XII a2). Whole body with 69 annuli. Annulation: I and II fused; III and IV bi-annulate; V–XXIV tri-annulate; XXV bi-annulate, and XXVI, XXVII uni-annulate (Fig. 8C).

**Figure 9.**
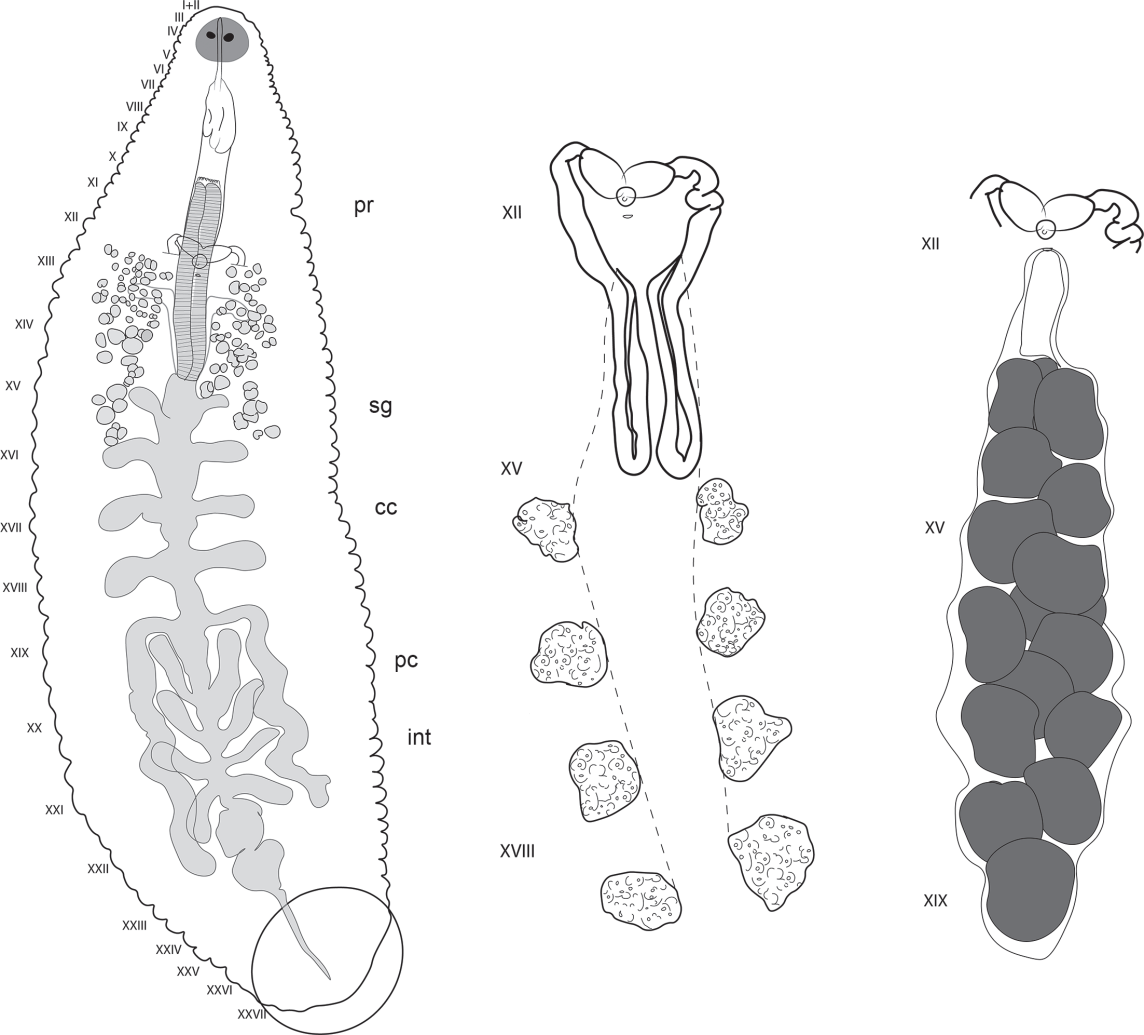
Internal morphology of a stained paratype specimen of *Helobdella gulloae* sp. nov. **A.** Digestive tract; **B.** Male reproductive system; **C.** Male and female reproductive systems. Abbreviations: pr = proboscis, sg = salivary glands, cc = crop caeca, pc = post-caeca, int = intestine.

***Internal morphology*.** Proboscis straight, not recurved, 1.52 length, extending from X to XIV. Salivary glands diffuse into parenchyma between XII and XV; ductules not forming bundle, insert independently into base of proboscis. Esophagus short, from at XVa1 to XV a2. Crop with five pairs of caeca; first four pairs digitiform and laterally directed; last pair with descending sinuous path or post-caeca, from XIX to XXII (Fig. 9A). Intestine with four pairs of digitiform caeca, the first three pairs directed anteriorly and last pair directed posteriorly. Testisacs in four intersegmental pairs, the first pair between XV/XVI, last pair XVIII/XIX. Ejaculatory ducts reaching the first pair of testisacs or XV/XVI (Fig. 9B). Ovisacs saccular, reaching XIX when it is filled (Fig. 9C).

***Reproductive information*.** No specimens were observed with spermatophores attached to the body wall, nor were any specimens found carrying eggs or young leeches on the ventral surface.

##### Etymology.

The species is named in honor of the Argentinian biologist Dr. Betina Sandra Gullo, in recognition of her significant contributions to the knowledge of South American leeches, in particular those within the genus *Helobdella*.

##### Remarks.

The morphological characteristics of *Helobdella gulloae* sp. nov. are consistent with those diagnostic of the genus (Sawyer 1986; Siddall and Borda 2003); including their dorsoventrally flattened body, gonopores separated by a single annulus, one pair of cephalic eyespots and the absence of esophageal organs (bacteriomes). The presence of multiple, longitudinal dorsal stripes and metamerically arranged papilla clearly support its inclusion in the “*triserialis*” series sensu Sawyer 1986.

*Helobdella gulloae* can be differentiated from other species of the “*triserialis*” series by difference of patterns of dorsal pigmentation and papillae distribution. *Helobdella papillata* has three rows of papillae restricted to the posterior third of dorsum (XIX or XX), while in *H. gulloae* sp. nov., dorsal rows of papillae begin at X. Additionally, *H. papillata* lacks dorsal pigmentation. In *H. lineata*, dorsal papillae are irregularly scattered, while in *H. gulloae* sp. nov. dorsal papillae are regularly arranged. *Helobdella transversa* lacks both longitudinal pigmented stripes and papillae (Sawyer 1972; Light and Siddall 1999), clearly contrasting with *H. gulloae* sp. nov. *Helobdella lineata* exhibits 12–14 longitudinal stripes on the dorsal surface, whereas *H. gulloae* sp. nov. display ~37. *Helobdella fusca* and *Helobdella virginiae* are characterized by irregularly arranged dorsal spots, clearly contrasting with the metameric pattern characteristic of *H. gulloae* sp. nov. (Table 2).

*Helobdella gulloae* sp. nov. is morphologically and phylogenetically (see below) closely related to *H. papilloprocta* sp. nov. The presence of two prominent papillae adjacent to the anus in *H. papilloprocta* sp. nov. appears to be a consistent and reliable character to separate both species. Furthermore, *H. gulloae* sp. nov. seems to be the only species of the “*triserialis*” series with only four pairs of testisacs, contrary to five or six pairs, which are more common in this genus.

The *cox*1 genetic distance, calculated under the K2P substitution model, between *H. papilloprocta* sp. nov. and *H. gulloae* sp. nov. is 3.33%. This value is comparable to the interspecific distance found between other sister species of *Helobdella*, such as *H. austinensis* and *H. virginiae* or *H. socimulcensis* and *H. farmeri*. The remaining pairwise genetic distances are presented in Table 3.

**Table 3.** *Cox*1 genetic distances among *Helobdella* species under the Kimura 2-parameter model. New species are indicated in bold.

Taxon	1	2	3	4	5	6	7	8	9	10	11	12	13	14	15	16
(1) *Helobdella europaea*	0.443															
(2) *Helobdella socimulcensis*	3.949	0.330														
(3) *Helobdella triserialis*	11.060	11.746	NA													
**(4) *Helobdella gulloae* sp. nov.**	11.000	11.584	8.522	NA												
**(5) *Helobdella papilloprocta* sp. nov.**	10.596	12.081	9.070	3.334	0.000											
(6) *Helobdella* cf. *socimulcensis Chiapas*	13.669	14.987	10.807	11.824	11.843	NA										
(7) *Helobdella fusca*	16.904	17.555	16.058	17.193	16.800	16.414	NA									
(8) *Helobdella austinensis*	18.919	19.621	19.105	18.725	18.879	17.042	22.060	0.483								
(9) *Helobdella virginiae*	17.094	17.568	16.950	17.579	17.704	17.009	21.409	3.789	NA							
(10) *Helobdella lineata*	18.305	18.225	18.497	18.594	18.270	16.664	20.985	11.771	12.078	NA						
(11) *Helobdella papillata*	17.414	18.047	17.254	17.748	17.143	15.874	18.742	9.745	10.305	6.853	NA					
(12) *Helobdella transversa*	17.331	18.366	18.089	18.620	17.634	16.693	20.302	10.286	10.305	8.334	4.753	NA				
(13) Helobdella cf. robusta	17.131	18.377	17.034	18.186	17.094	15.352	20.049	8.537	9.438	6.261	4.946	5.811	1.276			
(14) *Helobdella robusta*	19.880	20.578	18.066	19.324	18.093	17.541	20.329	11.917	12.041	7.013	8.085	10.605	7.087	NA		
(15) *Helobdella farmeri*	4.851	2.884	12.397	10.726	11.579	14.156	16.557	19.765	17.830	19.063	18.618	18.591	18.350	20.284	NA	
(16) *Helobdella* cf. *socimulcensis Jalisco*	3.894	3.230	11.614	11.275	11.665	13.623	17.606	18.854	16.914	17.703	17.453	17.850	18.493	20.017	4.105	NA

## ﻿Discussion

Based on morphology, we describe two new species of *Helobdella* of the “*triserialis*” series. Both species differ in both dorsal coloration pattern and annulation. Also, their disparate geographic distribution allows us to consider both as independent species. *Helobdella papilloprocta* sp. nov. possesses a pair of papillae next to the anus. Additionally, *H. gulloae* sp. nov. differs from *H. papilloprocta* sp. nov. by possession of four pairs of testisacs instead of five (Table 2). Both species are sister to each other and have a *cox*1 genetic distance of 2.8%, a value that might be low, but they are clearly different in both.

In addition to the two new species of the genus *Helobdella* described here, *H. austinensis* and *H. europaea* were recorded in Mexico for the first time. *Helobdella austinensis* was originally described from Austin, Texas, USA and here, we add three Mexican states to its geographic distribution: Nuevo León, Tamaulipas, and Veracruz. *Helobdella europaea*, on the other hand, is well known for its invasive ability, and here, we include Temixco, Morelos, Mexico, as a new locality for its distribution (Suppl. material 1).

With the description of two new species of *Helobdella* from Mexico and the two new country records provided here, a total of ten species of the genus are known in the country: *H. elongata*, *H. atli*, *H. temiscoensis*, *H. socimulcensis*, *H. virginiae*, *H. octatestisaca*, *H. papillanis*, *H. gulloae*, *H. austinesis*, and *H. europaea*.

## ﻿Conclusion

*Helobdella* is the most speciose leech genus worldwide, with ~80 named species. The majority of this diversity occurs in the New World, especially in the Neotropics, where it is expected that new species remain to be described. In this study, we provide the morphological characterization of two sister species that can be distinguished based on a combination of both morphology and DNA sequences, thereby contributing to our understanding of leech diversity.

## Supplementary Material

XML Treatment for Helobdella

XML Treatment for Helobdella socimulcensis

XML Treatment for Helobdella papilloprocta

XML Treatment for Helobdella gulloae

## ﻿Additional information

### Use of AI

No use of AI was reported.

### Author ORCIDs

Gerardo Torres-Carrera https://orcid.org/0000-0002-9229-1321

Lucas Duarte-De Lima https://orcid.org/0000-0001-6017-8327

Alejandro Oceguera-Figueroa https://orcid.org/0000-0002-5514-9748

### Data availability

All of the data that support the findings of this study are available in the main text or Supplementary Information.

## Supplementary materials

Supplementary material 1 *Helobdella austinensis* and *Helobdella europaea* Data type docx Explanation note **table S1**: Metadata associated with new records of two *Helobdella* species in Mexico. **fig. S1**: *Helobdella austinensis* from Sombreretillo, Nuevo León, Mexico. **A.** Dorsal view; **B.** Ventral view; **C.***Helobdella europaea* from Temixco, Morelos Mexico, SEM microphotograph. Scale bars: 2 mm. https://binary.pensoft.net/file/1478321This dataset is made available under the Open Database License (http://opendatacommons.org/licenses/odbl/1.0/). The Open Database License (ODbL) is a license agreement intended to allow users to freely share, modify, and use this Dataset while maintaining this same freedom for others, provided that the original source and author(s) are credited.Gerardo Torres-Carrera, Lucas Duarte-De Lima, Alejandro Oceguera-Figueroa
