# Supplementary material for: ﻿Description of two new species of freshwater leeches of the genus Helobdella (Hirudinea, Glossiphoniidae) from Mexico, with a redescription of Helobdella socimulcensis (Caballero, 1931)
Source: Zookeys. 2025 Nov 26;1261:141–64. doi: 10.3897/zookeys.1261.162279 (PMC12676574; doi:10.3897/zookeys.1261.162279)
Supplement: Supplementary material 1 — Helobdella austinensis and Helobdella europaea [file zookeys-1261-141_article-162279__-s001.docx]

Supplementary file

**Supplementary Table S1.** Metadata associated with new records of two *Helobdella* species in Mexico

| Taxon | Locality | Coordinates | CNHE number | # specimens |
| --- | --- | --- | --- | --- |
| *Helobdella austinensis* | Sombreretillo, Nuevo Leon | 26°18'46.2’’N; 99°57'21.9"W | 12857 | 2 |
|  | Balneary Gómez Farias, Tamaulipas | 22°59'32.9”N; 99°08'37.4"W | 12858 | 5 |
|  | Las Lajas, Veracruz | 21°17'26.0"N; 97°46'34.0"W | 12300 | 20 |
| *Helobdella europaea* | Temixco, Morelos | 18°5'00’’N; 99°13'57"W | 12301 | 1 |

**Supplementary Figure S1**. *Helobdella austinensis* from Sombreretillo, Nuevo León, Mexico. **A**. Dorsal view (scale bar: 2 mm); **B.** Ventral view; **C.** *Helobdella europaea* from Temixco, Morelos Mexico, SEM microphotograph. Scale bars: 2 mm.
